# Cholesterol and prevention of atherosclerotic events: limits of a new frontier

**DOI:** 10.1590/S1518-8787.2017051006416

**Published:** 2016-12-19

**Authors:** Luís Eduardo Teixeira de Macedo, E Faerstein

**Affiliations:** I Departamento de Epidemiologia. Instituto de Medicina Social. Universidade do Estado do Rio de Janeiro. Rio de Janeiro, RJ, Brasil

**Keywords:** Atherosclerosis, prevention & control, Early Diagnosis, Dyslipidemias, diagnosis, Hypercholesterolemia, epidemiology, Cardiovascular Diseases, prevention & control

## Abstract

Control of atherosclerotic cardiovascular disease – a highly prevalent condition and one of the main causes of mortality in Brazil and worldwide – is a recurrent subject of great interest for public health. Recently, three new guidelines on dyslipidemia and atherosclerosis prevention have been published. The close release of these important publications is a good opportunity for comparison: the Brazilian model has greater sensitivity, the English model does not work with risk stratification, and the American model may be overestimating the risk. This will allow reflection on current progress and identification of controversial aspects which still require further research and debate. It is also an opportunity to discuss issues related to early diagnosis and its efficiency as a preventive strategy for atherosclerotic disease: the transformation of risk into disease, the gradual reduction of cut-off points, the limitations of the screening strategy, and the problem of overdiagnosis.

## RESUMO

O controle da doença cardiovascular aterosclerótica - morbidade de alta prevalência e uma das principais causas de mortalidade no Brasil e no mundo – continua sendo tema de grande interesse para a Saúde Pública. Recentemente, três novas diretrizes sobre dislipidemia e prevenção da aterosclerose foram divulgadas. A convergência no tempo dessas importantes publicações constitui boa oportunidade para sua comparação: o modelo brasileiro tem maior sensibilidade, o inglês não trabalha com risco estratificado e o norte-americano parece estar superestimando o risco.Isso permitirá reflexões acerca dos avanços que já foram alcançados e identificação de aspectos ainda controversos, que seguem exigindo novas pesquisas e debates. É também uma oportunidade para discutir questões relacionadas ao diagnóstico precoce e sua eficiência como estratégia preventiva da doença aterosclerótica: as transformações do risco em doença, a diminuição progressiva de pontos de corte, as insuficiências da estratégia de rastreamento e o problema do sobrediagnóstico.

## INTRODUCTION

Cardiovascular disease (CVD) mortality has increased worldwide as a result of population growth and aging. In 2013, it accounted for almost a third of all deaths^3^. However, age-standardized mortality has declined significantly since the 1990s^3^.

In Brazil, CVD are also the leading cause of death. Nevertheless, they fell 31% between 1996 and 2007^20^. In the last 40 years, cerebrovascular disease was responsible for more deaths than coronary heart disease, probably due to the increased prevalence of high blood pressure, but with a declining trend in the 1980s and 1990s^11^.

In 2010, physical inactivity and inadequate diet accounted for 10% (95%CI 9.2–10.8) of global losses in disability-adjusted life years (DALY), which is the sum of years lost due to premature death and years lost due to disability, adjusted to their severity weight. High blood pressure accounted for a loss of 7% (95%CI 6.2–7.7), and smoking for 6.3% (95%CI 5.5–7.0)^10^.

The first attempt to overcome the single-factor view of atherosclerotic CVD risk was in New Zealand in 1993. But the major advance in the joint use of risk factors came in 1998 with the publication of an article by the Framingham Heart Study team. Thus, it was possible to estimate the absolute risk of developing coronary heart disease per decade of life according to gender and age, systolic blood pressure, total cholesterol, HDL-cholesterol, diagnosis of diabetes, and knowledge of smoking habits^12^.

Recently, three new guidelines on dyslipidemia and atherosclerosis prevention have been released. In October 2013, the Brazilian Society of Cardiology published “V Diretriz sobre Dislipidemias e Prevenção da Aterosclerose” (V Guideline on Dyslipidemias and Atherosclerosis Prevention)^25^. One month later, the American College of Cardiology and the American Heart Association published the “Guideline on the Assessment of Cardiovascular Risk”^4^. Finally, in September 2014 in England, the National Institute for Health and Care Excellence published an update on the modification of serum lipids aimed at primary and secondary prevention of cardiovascular diseases^a^.

The new guidelines recommend different therapeutic interventions, but they are all based on individual absolute risk, estimated from different multivariate statistical models of risk prediction. Our Table compares the main characteristics of each one of these guidelines. There are several aspects worth considering.

**Table:** Summary of the main characteristics of new national and international guidelines for cholesterol and atherosclerotic disease.

	Guideline			

	SBC V Guideline		ACC/AHA Guideline	NICE
Country	Brazil		United States	England
Release	October/2013		November/2013	September/2014
Age of assessed individuals	Adults > 30 years		Adults > 20 years	Adults > 40 years
Period related to absolute risk	10 years or throughout life^a^		10 years or throughout life^b^	10 years^c^
Risk prediction tool	Global Risk Score		Pooled Cohort Equations	QRISK 2
Outcomes related to risk	Acute Myocardial Infarction (AMI); Stroke; Peripheral Vascular Disease; or Congestive Heart Failure		AMI; Death by Coronary Heart Disease; fatal and non-fatal stroke	AMI; Death by Coronary Heart Disease; fatal and non-fatal stroke
Risk level:	Men	Women		
High	≥ 20.0%	> 10.0%	> 7.5%	No risk stratification^d^
Intermediate	5.0% a 19.0%	5.0% a 10.0%	5.0% a 7.5%	
Low	0% a 4.0%	0% a 4.0%	< 5.0%	
Sugested therapy	Antilipemics		Statins^f^	Statins^f^
Previous LDL-cholesterol limits	Low risk: < 160 mg/dl^e^		Low risk:< 160 mg/dl	Secondary prevention only
	Medium risk: < 130 mg/dl^e^		Medium risk:< 130 mg/dl	
	High risk: < 100 mg/dl^e^		High risk: < 100 mg/dl	
Current LDL-cholesterol limits	Low risk: individualized limit		No limits	Secondary prevention only
	Medium risk: < 100 mg/dl^e^			
	High risk: < 70 mg/dl^e^			

ACC: American College of Cardiology; AHA: American Heart Association; NICE: National Institute for Health and Care Excellence; QRISK: QRESEARCH Cardiovascular Risk Algorithm

^a^ Indicated for individuals with low risk over 10 years and aged > 45.

^b^ Indicated for individuals with low risk over 10 years; calculated for a 30-year period.

^c^ Risk also presented for longer periods, with continuous variable.

^d^ Uses 10,0% and 20,0% of absolute risk as reference point for therapy indication.

^e^ Stricter limits compared to “IV Diretriz sobre Dislipidemias e Prevenção da Aterosclerose da Sociedade Brasileira de Cardiologia” (Afiune Neto A, Souza AD, Lottenberg AMP, Chacra AP, Faludi AA, Loures-Vale AA, et al. IV Diretriz Brasileira Sobre Dislipidemias e Prevenção da Aterosclerose do Departamento de Aterosclerose da Sociedade Brasileira de Cardiologia. *Arq Bras Cardiol.* 2007;88(Supl I):1-19).

^f^ Increased therapy intensity for higher risks.

Firstly, the outcomes for which the risk is estimated differ. Simply expanding the clinical possibilities for which the risk is calculated increases the likelihood that they are present, and this will always be translated as greater absolute risk.

A second aspect worth considering is the relationship between age and risk levels. It is known that absolute risk of atherosclerotic disease increases with age; on the other hand, the relative weight of the other risk factors in the composition of this absolute risk decreases. Individually, age can select the groups with the highest individual risk. Regarding coronary and cerebrovascular events, a cut-off point of 55 years could be used to identify 96% of future fatalities^23^. In other words, the impact of lowering LDL-cholesterol on the absolute risk reduction of an atherosclerotic event decreases with age.

In the three guidelines, absolute risk is presented in levels graded as low, medium and high. However, both the variables that make up the equations and the limits between each risk range differ between the guidelines. Therefore, the estimated risk and, consequently, the recommended intervention for a same person may differ according to the guideline chosen.

It is common for clinical trial outcomes to be presented to practitioners in terms of relative risk reduction. However, decision-making requires absolute measures, i.e., absolute risk reduction (ARR)^19^. An example is shown by comparing screening strategies that generate the same relative risk reduction of 20% in diseases with different mortality rates. The first disease has a 5% mortality rate, and screening would reduce it to 4% (20% of 5%). The absolute risk reduction is 1% (5% minus 4%), and the number of people needed for screening^18^ is 100 (1 divided by 1% [1/ARR]). For every 100 people not screened, five will die, and for every 100 people screened, four will die. That is, for every 100 people screened, it is possible to save a life. The second disease has a lower mortality rate of 0.5%, and therefore screening will reduce mortality to 0.4% (20% of 0.5%). The absolute risk reduction is 0.1% (0.5% minus 0.4%), and the number of people needed for screening is 1,000 (1 divided by 0.1%). In this case, 1,000 people would have to be screened to avoid one death^19^.

The Framingham Study has a long history of producing multivariable predictive risk models. The use of tables associating the occurrence of risk factors with the given score, which will result in a total score, stems from the observation of the weight each factor has in these equations. If, on the one hand, the resource contributes to operational ease, on the other it oversimplifies the multifactorial nature of atherosclerotic disease and may generate loss of information and precision in the results. In addition, the ethnic composition of the study population is quite different compared to the rest of the world and to Brazil in particular^22^.

The validity of using these risk scores can also be questioned. The assimilation of a predictive model for clinical practice requires the prior performance of four distinct evaluations. First, identification of the variables that will compose the model. Then, validation of the tool in the population where it was generated, when investigating its capacity to distinguish cases from non-cases. Next, validation in external populations, or calibration, when comparing calculated risks with observed risks. And, finally, analysis of the impact of the use of the tool in actual clinical practice^1^. In the case of the US guidelines, if on the one hand there was a concern with recommending only those interventions already evaluated in randomized clinical trials, on the other the same criterion was not applied in selecting the risk prediction model. Indeed, application of the Pooled Cohort Equation in the population of the Women’s Health Initiative, Women’s Health Study and the Physician’s Health Study indicated overestimation of cardiovascular risks, which reached more than 150%^17^.

These guidelines are not protocols for the treatment of dyslipidemias or atherosclerosis, but for the prevention of ischemic events resulting from atherothrombotic phenomena, based on control of serum cholesterol levels. Thus, it is relevant to go back to the conceptual basis of preventive medicine systematized by Leavell and Clark^8^. The natural history model proposed by the authors presents the process of illness in distinct phases. Initially, there is a phase of susceptibility, when exposure to risks and induction of disease occurs. Then the disease begins, but without manifest signs or symptoms, in a phase called subclinical, or latency period. The next step is symptomatic disease, also called the clinical phase. The idea of predisease originated from early 20th century oncology^9^. The term prediabetes appeared in the medical literature around 1940^6^; prehypertension, in turn, is a more recent term, introduced in 2003^7^. Thus, the predisease phase precedes the disease stage – including subclinical. That is, it concerns events that happen still in the induction period.

A predisease does not always progress to the next stage. Among people with prehypertension, 20% to 30% stabilize their blood pressure levels over time, without undergoing any intervention. Likewise, individuals with fasting blood glucose levels between 100 mg/dl and 110 mg/dl, conceptually in a prediabetes state, when monitored for up to 20 years did not present a higher risk than those of individuals with normal glycaemia for both the development of retinopathy, nephropathy or neuropathy and the occurrence of major cardiovascular events^7^.

Subclinical atherosclerosis comprises states ranging from initial reactions to risk exposure, especially endothelial dysfunction, to the presence of calcified, inflamed, and unstable atheromatous plaques (Figure). It makes epidemiological sense to observe that, in a causal chain, the more distal the exposure, the lower its strength of association with the outcome^2^. That is, the strength of association between exposure to hypercholesterolemia and acute myocardial infarction should be lower than that observed between the calcified atheromatous plaque and infarction.

**Figure:**
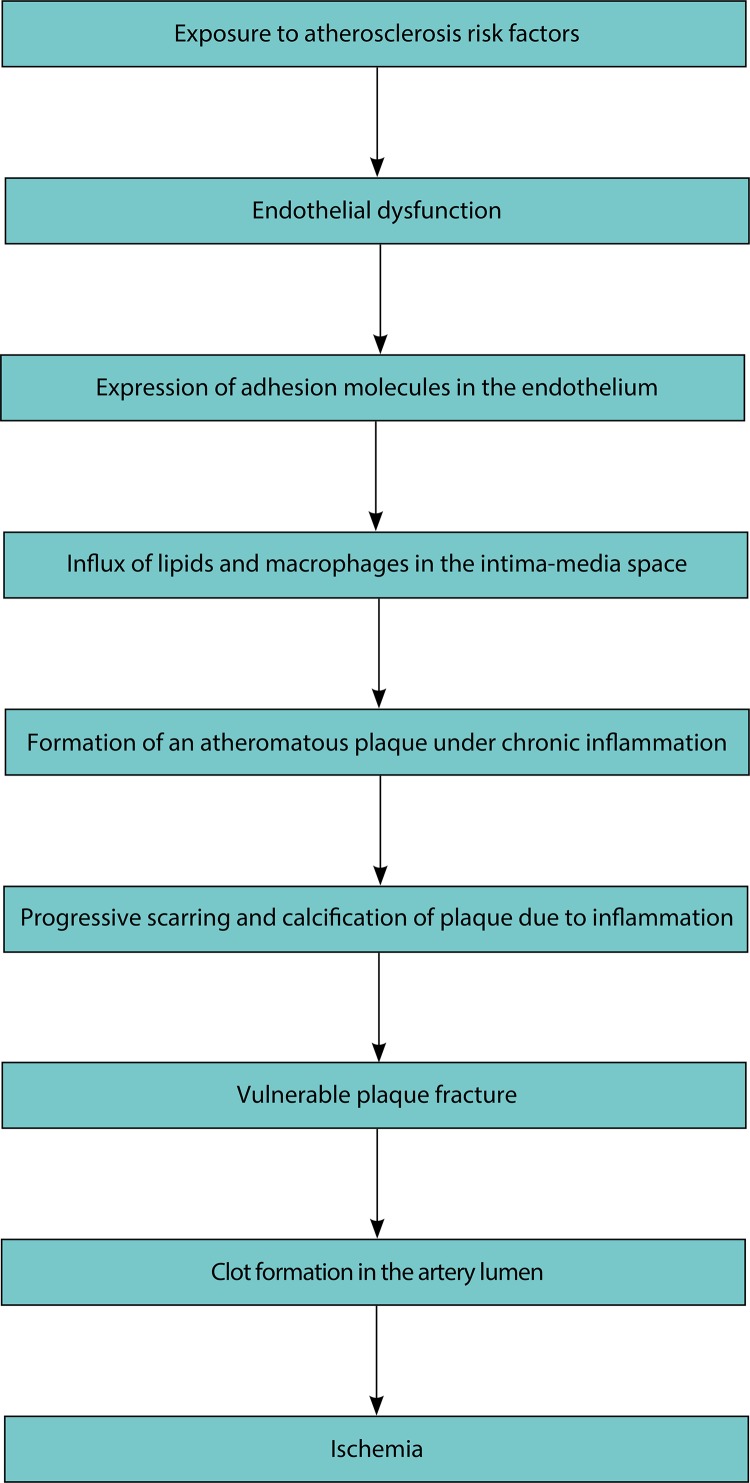
Sequence of events in the pathophysiology of atherosclerotic cardiovascular disease.

Thus, a question arises: at what point in the natural history of atherosclerosis will the detection of abnormalities decrease the likelihood of advanced disease or death? If early diagnosis of a disease in the clinical phase is always desired, the same may not be true regarding the previous phases of atherosclerotic disease. At least, not until randomized clinical trials have assessed the impact of therapeutic intervention based on the screening of each one of these stages of the pathophysiological chain and proven the effectiveness of this strategy.

There are two different prevention strategies: the modification of population risks and the modification of individual risks. The former stems from the observation that the variability of a given characteristic within a specific population – serum cholesterol, for example – tends to form a unimodal continuous distribution^14^. The population prevention strategy aims to shift the whole distribution of risk frequency in a favorable direction. To this end, it is necessary to conduct interventions that modify the behavior of the whole society. It is a strategy with a longer reach in the causal chain and of more lasting effect^19^.

The prevention strategy that focuses on high individual risk interferes with a smaller part of the total population and offers cost-effective interventions. It is narrow in scope, since it does not modify adverse behaviors or unjust social structures. It has a palliative, local and temporary effect and little capacity to reduce the population burden of disease^19^.

Screening is a prevention strategy that seeks patients in an asymptomatic subpopulation with high individual risk to impose treatment that modifies the natural history of the disease^16^. The guidelines in question start out from this procedure, but once the risk is stratified, treatment is soon proposed. However, two steps are missing in this process: performance of screening and diagnostic tests. Proposing treatment for the entire high-risk group will inevitably increase the occurrence of false-positives and overdiagnosis.

The medical literature has modified the limits that define important diseases related to atherosclerosis, with great impact in the calculation of their prevalence. In the United States, the decrease in requirements regarding total serum cholesterol, from 240 mg/dl to 200 mg/dl, added 42,647,000 people to the patient group (+86%). For every 100 people diagnosed with dyslipidemia based on the new limits, 78 will never progress to the clinical phase of the disease. The diagnosis and treatment of this population group are called, respectively, overdiagnosis and overtreatment^24^.

However, it is not possible to differentiate individuals whose clinical situation will reach clinical significance from those who will never cross that border. It is possible to recognize overdiagnosis by comparing the incidence of clinical disease among screened and unscreened populations; by identifying subclinical disease during necropsy studies; and by the fact that there are poorly calibrated predictive models that overestimate morbidity and mortality^24^.

Excessive use of certain screening and diagnostic tests is an important component of health care cost in many countries – in the United States, over $2.2 trillion in 2008. The American College of Physicians has been involved in the effort to differentiate the value of an intervention based on the identification of its risks, costs and benefits^15^. More than US$200 billion may be wasted annually in the United States with unnecessary treatment^13^, where preventable medical errors committed in hospital environments are now the sixth leading cause of death^21^.

It is essential for Brazilian health researchers and practitioners to also debate the excesses involving asymptomatic individuals in the name of prevention, based on the belief – not necessarily always true – that the earlier the diagnosis, the better.

## Footnotes

a National Institute for Health and Care Excellence. Lipid modification: cardiovascular risk assessment and the modification of blood lipids for the primary and secondary prevention of cardiovascular disease. London: National Institute for Health and Care Excellence; 2008 [cited 2015 Feb 27]. (NICE Guidelines CF67). Available from: http://www.nice.org.uk/guidance/cg181/resources/guidance-lipid-modification-cardiovascular-risk-assessment-and-the-modification-of-blood-lipids-for-the-primary-and-secondary-prevention-of-cardiovascular-disease-pdf
